# Tremor Ataxia With Central Hypomyelation Phenotype Related to a Recurrent POLR3A Mutation in Six Unrelated Tunisian Families

**DOI:** 10.1002/mgg3.70007

**Published:** 2024-10-22

**Authors:** Ichraf Kraoua, Maha Jamoussi, Cyrine Drissi, Lilia Kraoua, Séverine Drunat, Hanene Benrhouma, Thouraya Ben Younes, Sonia Nagi, Sonia Abdelhak, Odile Boespflug Tanguy, Ilhem Ben Youssef‐Turki, Mediha Trabelsi, Imen Dorboz

**Affiliations:** ^1^ LR18SP04, Department of Child and Adolescent Neurology Faculty of Medicine of Tunis, National Institute Mongi Ben Hmida of Neurology, University of Tunis El Manar Tunis Tunisia; ^2^ Department of Neuroradiology, Faculty of Medicine of Tunis, National Institute Mongi Ben Hmida of Neurology University of Tunis El Manar Tunis Tunisia; ^3^ Department of Congenital and Hereditary Diseases, Faculty of Medicine of Tunis, Charles Nicolle Hospital University of Tunis El Manar Tunis Tunisia; ^4^ Génétique Moléculaire, DMU BioGeM, APHP, Hôpital Robert Debré Paris France; ^5^ INSERM UMR1141, Hôpital Robert Debré, Université Paris Cité Paris France; ^6^ LR11IPT05, Laboratory of Biomedical Genomics and Oncogenetics Pasteur Institute of Tunis, University of Tunis El Manar Tunis Tunisia; ^7^ Service de Neuropédiatrie, Centre de Reference LEUKOFRANCE, APHP, Hôpital Robert Debré Paris France

**Keywords:** ataxia, hypomyelination, MRI, *POLR3A* gene

## Abstract

**Background:**

POLIII‐related leukodystrophies are a group of recently recognized hereditary white matter diseases with a similar clinical and radiological phenotype. No Tunisian studies have been published about POLIII‐related leukodystrophy due to *POLR3A* variants. The aim of this study was to contribute to the clinical, radiological, and genetic characterization of *POLR3A*‐related leukodystrophy in a Tunisian cohort.

**Methods:**

We report six cases of genetically confirmed *POLR3A*‐related leukodystrophy belonging to six unrelated Tunisian families, along with a review of previously published pediatric cases.

**Results:**

All patients were born to consanguineous marriages and originated from the North or the Center of Tunisia. Age at onset varied between 15 months and 6 years.

The clinical phenotype was similar in all patients with cerebellar ataxia, tremor, and nystagmus being the key features. Brain imaging showed diffuse hypomyelination in all patients with progressive cerebellar atrophy in three patients.

Molecular analysis identified the same bi‐allelic NM_007055.4:c.2011T>C; p.(Trp671Arg) variant in the *POLR3A* gene in all patients.

**Conclusion:**

We hypothesize a founder effect for the identified variant given its recurrence in six unrelated individuals with a similar clinical phenotype. Given the apparent genetic homogeneity of Tunisian *POLR3A* patients, the recurrent variant should be directly targeted. This should facilitate diagnosis in index patients, and genetic counseling.

## Graphical abstract

We report on six unrelated families with the same *POLR3A* mutation exhibiting a similar clinical phenotype. We hypothesize a founder effect for this recurrent mutation and suggest that it should directly targeted in suspected cases, which should facilitate diagnosis in index patients, and genetic counseling.

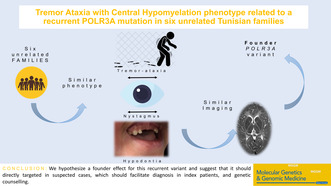

## 1 Introduction

POLIII‐related leukodystrophy is a rare but not uncommon form of hypomyelinating leukodystrophy, mostly affecting children, which is due to variants in the subunits of the RNA polymerase III (POLIII). Five main genes are involved in the disease: *POLR3A* (OMIM 607694), *POLR3B* (OMIM 614381), *POLR1C* (OMIM 616494), and more recently discovered, *POLR3D* (OMIM 187280) and *POLR3K* (OMIM 617456) (Daoud et al. 2013; Dorboz et al. 2018; Macintosh et al. 2023; Pelletier et al. 2021).

*POLR3A* was the first gene reported in French‐Canadian families affected by the tremor‐ataxia with central hypomyelination (TACH) (Bernard et al. 2011).

A broader clinical phenotype has been subsequently reported with a continuum of overlapping neurological signs (tremor, ataxia, dystonia spasticity) with central hypomyelination and non‐neurological signs, mainly, hypo/oligodontia, ocular symptoms, and hypogonadotropic hypogonadism (Bernard et al. 2011).

The recent genetic characterization of this group of diseases has led to genotype–phenotype correlations such as the c.1909 + 22G4A variant combined with a second, “variable” *POLR3A* variant correlating with juvenile‐adult‐onset spastic ataxia with superior cerebellar peduncle hyperintensity and spinal cord atrophy (Infante et al. 2020). However, unusual presentations have also been reported (Harting et al. 2020).

The phenotype of these diseases remains heterogeneous and this is probably due to genetic variability. In Tunisia, POLIII‐related leukodystrophies caused by *POL3RA* variants have not been reported before.

The aim of this study was to contribute to the clinical, radiological, and genetic characterization of POLIII‐related leukodystrophies caused by *POL3RA* variants, based on the analysis of six cases belonging to six unrelated Tunisian families with a review of previously published pediatric onset cases (with an age of onset before 18 years of age).

## 2 Patients and Methods

### 2.1 Patients and Data Collection

Patients from this clinical series were recruited from the Department of Pediatric Neurology, at the National Institute of Neurology, Mongi Ben Hamida of Tunis, between 2005 and 2023. Patients were examined by an expert in leukodystrophies and metabolic disorders. In terms of screening, all patients underwent routine blood tests, brain magnetic resonance imaging (MRI), and other investigations including electroneuromyography. Raven's progressive matrices number 47 were used for cognitive assessment in our patients.

### 2.2 Molecular Investigation

Blood samples were collected after informed consent of all families and approval from the local ethics committee.

#### 2.2.1 Panel Screening

For four patients, genomic DNA was screened for variants, using NGS panel testing 154 leukodystrophies and leukoencephalopathies causing genes (NextSeq500 Illumina) in LEUKOFRANCE reference center in Paris, France. The list of genes included in the panel and associated diseases can be found in https://www.orpha.net/consor/cgi‐bin/ClinicalLabs_Search.php?lng=EN&data_id=118000&search=ClinicalLabs_Search_Simple&data_type=Test&title=Diagnostic%20des%20leucodystrophies%20Panel&MISSING%20CONTENT=Diagnostic‐des‐leucodystrophies‐‐Panel‐. The library was prepared using Custom SureSelectQXT (GC_V3) Agilent. BWA algorithm was used to align genomic DNA sequence to human reference genome GRCh37 (hg19) with >99.5% coverage and minimum depth of 20X. Variants were classified using Bench lab NGS Cartagenia v5.0.1.

#### 2.2.2 Sanger Sequencing

Exon 15 of *POLR3A* was analyzed in the department of Congenital and Hereditary diseases of Charles Nicolle Hospital in Tunis in the two remaining patients, targeting the variant identified by NGS in the four patients mentioned above. PCR amplification and direct DNA sequencing were performed. The Big Dye Terminator cycle sequencing kit (version 3.1, Life Technologies, Thermo Fisher Scientific, Carlsbad, CA, USA) and capillary electrophoresis on the SeqStudio genetic analyzer (Applied Biosystems, Thermo Fisher Scientific, Waltham, MA, USA) were used for DNA sequencing reaction. Sequence data were analyzed using Sequencing Analysis Software (version 6.0; Thermo Fisher Scientific, Waltham, MA, USA) and the NCBI reference sequence NM_007055.4.

### 2.3 Literature Review

We conducted a literature review via NIH PubMed database using the keywords: “POLR3 leukodystrophy,” “POLR3A,” and “4H leukodystrophy.”

The variant found was entered in the Clinvar database to verify its significance and search for other published cases.

Cases with unspecified clinical and radiological features (genetic characterization only), were excluded from the review. We estimated the frequency of each clinical and radiological feature in the resulting cohort.

## 3 Results

We included six patients (two females and four males), from unrelated families. Demographic, clinical, radiological, and genetic findings of the six cases are summarized in Table 1.

**TABLE 1:** Demographic, clinical, and genetic findings in our patients with *POLR3A*‐related leukodystrophy.

Patient/family	LD1	LD2	LD3	LD4	LD5	LD6
Current age (years)	13	22	12	10	10	07
Gender	F	F	M	F	M	M
Consanguinity	+	+	+	+	+	+
Geographic origin	Siliana (North West)	Sejnane (North East)	Bouhajla (Center)	Sidi Bouzid (Center)	Menzel Bourguiba (North East)	Jendouba (North West)
*POLR3A* gene variant NM_007055.4	c.2011T>C; p.(Trp671Arg)	c.2011T>C; p.(Trp671Arg)	c.2011T>C; p.(Trp671Arg)	c.2011T>C; p.(Trp671Arg)	c.2011T>C; p.(Trp671Arg)	c.2011T>C; p.(Trp671Arg)
Personal history	Unremarkable	Unremarkable	Unremarkable	Unremarkable	Unremarkable	Unremarkable
Family history	+ One brother died at the age of 5 years	+ 2 paternal cousins died at 30 and 15 years	−	−	+ Death at 3 days old of a younger sister	−
Age of onset	15 months	6 years	2 years	3 years	5 years	2 years
Psychomotor development						
Walking age acquisition	15 months	12 months	16 months	14 months	12 months	20 months
Language	Normal	Normal	Delayed	Normal	Normal	Delayed
Fine motor skills	Impaired writing, grasping, and manipulating objects	Initially normal	Impaired writing, grasping, and manipulating objects	Impaired writing, grasping, and manipulating objects	Impaired (writing)	Impaired writing, grasping, and manipulating objects
Reading	Adequate	Adequate	Impossible	Adequate	Impaired	Adequate
Comprehension	Adequate	Adequate	Impaired	Adequate	Impaired	Adequate
Behavior	Normal	Normal	Psychomotor agitation	Normal	Normal	Normal
Inaugural neurological symptoms	Unsteady gait (ataxia) initially followed by upper limb tremor at the age of 3 years	Unsteady gait (ataxia), dysarthria	Gait ataxia, upper limb and head tremor	Gait ataxia, upper limb and head tremor	Upper limb tremor followed by unsteady gait (ataxia)	Unsteady gait (ataxia) initially followed by upper limb tremor at the age of 6 years
Neuro‐ophthalmological manifestations						
Nystagmus	+	+	+	−	+	+
Supranuclear gaze palsy	−	−	+	−	−	−
Short‐sight (myopia)	−	−	−	−	+	−
Cataract	−	−	−	−	−	−
Neurological manifestations						
Pyramidal signs	−	−	+	−	−	−
Cerebellar ataxia	+	+	+	+	+	+
Tremor	+	−	+	+	+	+
Cerebellar dysarthria	+	+	+	+	+	+
Dystonia	+	+	++	−	+	−
Myoclonus	+	−	+	−	−	−
Epilepsy	−	−	−	−	−	−
Psychomotor regression	+	+	+	−	−	−
Associated signs						
Hypogonadotropic hypogonadism (low FSH, LH rates)	−	+	−	− Ovarian hypoplasia on abdominal ultrasound	+	+
Pubertal delay	−	+ no menarche	−	−	NA	NA
Small stature	−	−	−	−	+	+
Dental anomalies	−	−	+ delayed eruption	+ hypodontia	+ abnormal shape	+ hypodontia
Swallowing disorders	−	+ (19 years)	−	−	−	−
Outcome						
Bedridden	+ (15 years)	+ (19 years)	+ (11 years)	−	−	−
Cognitive decline/ language decline	+	+ (unable to communicate since the age of 17 years)	+ (no social contact at the age of 11 years)	+ (language decline, impaired communication)	+ (academic difficulties, irritability)	−
Worsening after an infection	+	−	+	+	−	−
Death	−	−	−	−	−	−
Investigations						
ENMG	Normal	Normal	Normal	Normal	Normal	Normal
Brain MRI (age) Change (0: normal, +: mild, ++: severe)	5 and 6.5 years Change +	11 and 16 years Change ++	7 years	5 years	10 years	6 years
Diffuse hypomyelination including U‐fibers	+	+	+	+	+	+
Internal capsule involvement (posterior limb)	−	+	−	+	−	−
Corpus callosum involvement	+	+	−	+	+	−
Cerebellar white matter hypersignal	+	+	−	+	−	+
Ventrolateral thalamus hyposignal	±	+	+	−	−	−
Cerebellar atrophy	±	+ (progressive worsening)	−	−	−	+
Corpus callosum atrophy	±	−	−	−	+	−

Abbreviations: ENMG: electroneuromyography; F: female; M: male; MRI: magnetic resonance imaging; NA: non applicable; +: present; −: absent.

All couples were consanguineous. Families originated from different parts of the country in the North West and the Center of Tunisia. Similar cases within the family were noted in two patients. Perinatal history was unremarkable in all patients.

Initial psychomotor development was normal in four patients. Two patients had delayed psychomotor milestones: one patient walked at the age of 20 months and had a delayed speech with a two‐word stage at the age of 24 months, and the other patient walked at the age of 16 months with first words at the age of 18 months.

Age of onset ranged between 15 months and 6 years. The clinical phenotype was homogeneous in our series with gait ataxia as a presenting symptom in five out of six patients. The remaining patient had upper‐limb tremor as a presenting symptom, followed by gait ataxia.

Neurological examination found a static and kinetic cerebellar syndrome in all patients. In addition, dystonia was found in four patients, myoclonus in two patients, and one patient exhibited pyramidal signs.

Five patients had nystagmus and one patient had vertical gaze limitation. On follow‐up, progressive worsening was noted in all patients and two patients lost their walking capacity at the age of 11 and 18 years. Cognitive decline was moderate but present in all patients.

Speech disorders were mainly due to cerebellar dysarthria, which was also present in all patients. An acute worsening of symptoms was reported after intercurrent infections in three patients.

**FIGURE 1:**
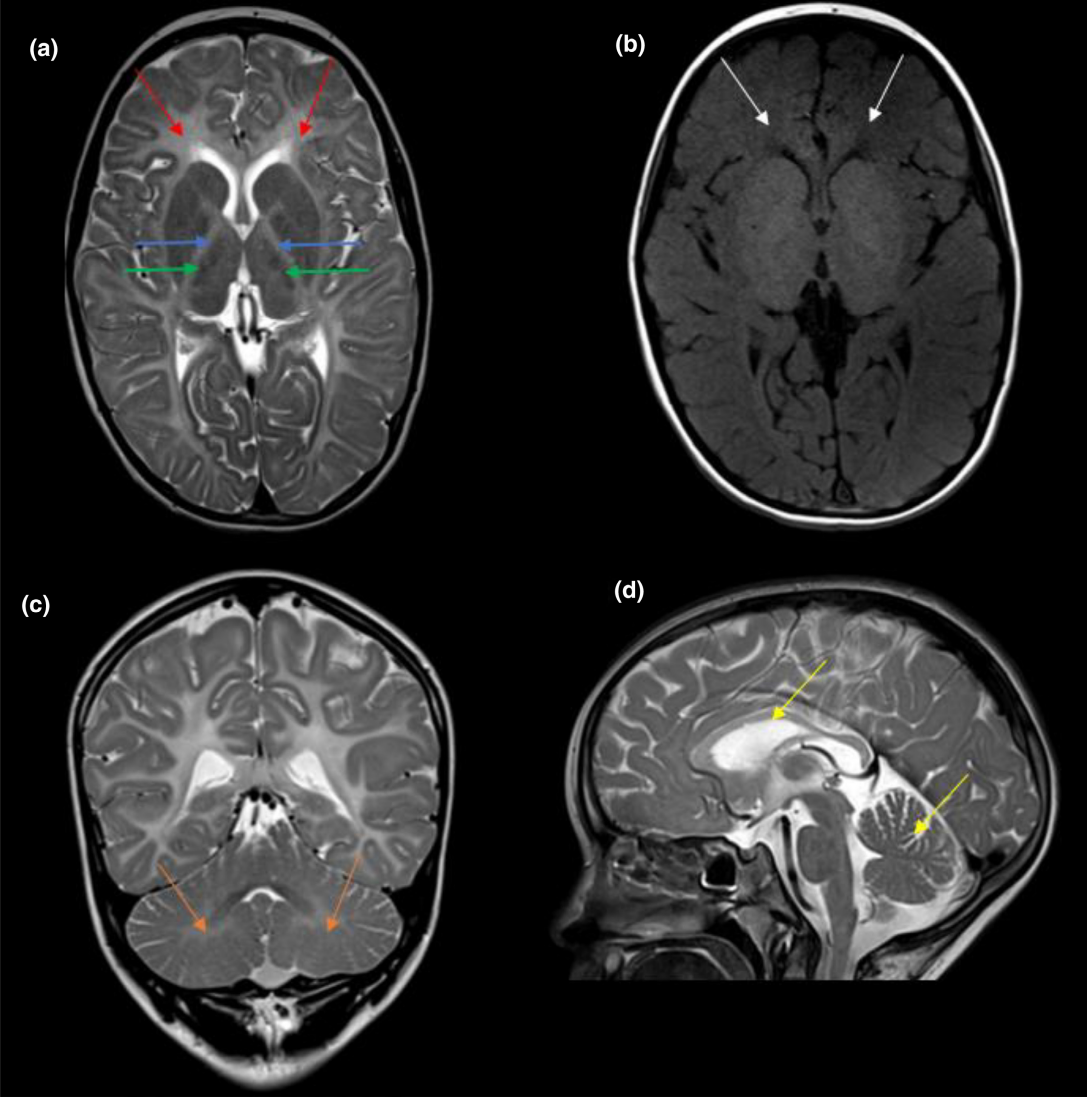
Brain MRI imaging of a five‐year‐old patient in our series (Patient 2). Axial T2‐weighted image (a) showing diffuse white‐matter hyperintensity with U‐fiber involvement (red arrows) and posterior limb of internal capsules involvement (blue arrows). A relative T2 hypointensity of the ventrolateral nuclei of the thalami can also be noted (green arrows). Axial T1‐weighted image (b) showing corresponding T1 isointensity in comparison with the cortical gray matter, compatible with a hypomyelinating process (white arrows). Coronal T2‐weighted image (c) showing diffuse hypomyelination including cerebellar white matter (orange arrows). Sagittal T2‐weighted image (d) showing moderate cerebellar and corpus callosum atrophy (yellow arrow). Also note hypomyelination involvement of the corpus callosum.

On brain MRI, all patients presented with diffuse supra‐tentorial hypomyelination, involving the periventricular and the subcortical region as well as U‐fibers (Figure 1). Corpus callosum involvement was noted in four patients, cerebellar white matter involvement in three patients, and posterior limb of internal capsule involvement in two patients. Cerebellar atrophy was noted in three patients and seemed to worsen on follow‐up imaging in two patients. Three patients had a relative T2 hyposignal in the ventrolateral nuclei of the thalami. Corpus callosum thinning was seen in two patients.

In terms of extra‐neurological signs, four patients had dental abnormalities, one female patient had pubertal delay, and two male patients had failure to thrive. Hypogonadotropic hypogonadism was noted in three patients (two males and one female), with very low serum FSH and LH rates.

Table 2 provides a comparative review of 77 reported cases of pediatric onset *POLR3A*‐related diseases, including our patients (Bernard et al. 2011; Daoud et al. 2013; Hiraide et al. 2020; Nikkhah and Rezakhani 2022; Saitsu et al. 2011; Shimojima et al. 2014; Tewari et al. 2018; Wolf et al. 2014; Wu et al. 2019).

**TABLE 2:** Main clinical and imaging features in reported patients with *POL3A*‐related leukodystrophy.

Reference	Nikkhah and Rezakhani (2022)	Hiraide et al. (2020)	Wu et al. (2019)	Tewari et al. (2018)	Wolf et al. (2014)	Vanderver et al. (2013)	Shimojima et al. (2014)	Bernard et al. (2011)	Saitsu et al. (2011)	Our cases	Total
Number of patients	1	3 (2 families)	1	1	43	1	1	19	1	6	77 patients
Age at last examination (years)	6	18	NA	5	3–40 (20)	36	29	NS	17	7–22	−
Gender	M	1M/2F	F	F	20M/23F	M	F	9M/10F	1M	3M/3F	36M/41F
Psychomotor delay	+	2/3	+	+	52% (54/103) of the whole cohort including *POLR3B*	−	+	7/19 (37%)	−	1/6	≈23%
					4/43 never able to walk unsupported						
Age of onset	2 years	10 months to 8 years	9 months	Neonatal	1–13 years (mean 4.3 years)	13 years	1 year	7 years (0–13 years)	4 years	15 months to 6 years	Neonatal to 13 years
Cerebellar syndrome	+	1/3	+	+	99%	+	+	19/19	+	6/6	**96%**
Tremor	−	1/3	−	+	NA	NA	+	11/19	+	5/6	≈55%
Oculomotor signs											
Nystagmus	NA	2/3	+	+	99%	+	+	3/19	−	5/6	≈71%
Vertical gaze limitation	NA	NA	NA	NA	20%	+	NA	6/19	−	1/6	−
Abnormal smooth pursuit	+	1/3	NA	NA	NA	+	NA	13/19	NS	−	−
Optic atrophy	−	NA	−	+	NA	NA	NA	4/19	−	0/6	−
Pyramidal signs	+	3/3	+	+	NA	+ with spastic paraplegia	+	19/19	−	1/6	≈82%
Dystonia	+	2/3	−	−	Few patients	−	−	−	NA	4/6	≈20%
Myoclonus	−	1/3	−	−	−	−	−	−	NA	2/6	≈4%
Language	Delayed	NA	Delayed + deterioration	Delayed	Deterioration	Dysarthria	Deterioration	NA	NA	Delayed 2/6	−
Swallowing problems/dysphagia	+	3/3	+	−	+ (% NS)	+	NA	7/19	NA	1/6	−
Mental retardation	+	3/3	+	NA	normal to learning difficulties	+	+	NA	+	NA	−
Cognitive regression	+	3/3	+	−	NA	+	+	19/19	−	3/6	≈85%
Epilepsy	+	0/3	+	−	19%	−	+	3/19	−	0/6	≈18%
Sphincter problems	NA	NA	NA	NA	NA	NA	NA	NA	NA	−	−
Neurological deterioration with infection	+	NA	+	NA	Adolescence—53%	NA	NA	NA	NA	3/6	−
Motor deterioration/wheelchair	NA	NA	NA	+	100% (1–33 years)	NA	+	12/19 (5–30 years)	+	2/6	−
Peripheral nerve involvement	−	NA	NA	−	NA	+ (hyporeflexia)	NA		−	−	−
Death	+ (age NS)	−	−	−	50% (6‐35 years)	+ (choking)	−	3/19 (21,28,36)	−	−	35%
Non neurological signs											
Myopia	−	NA	−	NA	87%	+	−	NA	+	1/6	≈52–87%
Cataract	−	NA	−	NA	3%	−	−	NA	NS	0/6	−
Dental abnormalities	−	2/3	+	+	87%	+	+ (delayed eruption)	15/19	−	4/6	≈76–87%
Hypogonadotropic hypogonadism	Failure to thrive‐NS	−	NA	−	81%	+	+	7/19	−	3/6	≈60%
Brain MRI											
Diffuse hypomyelination	−	0/3	+	+	100%	+	+	19/19	+	6/6	**97%**
Dot of posterior limb of internal capsule	−	0/3	−	+	13%	NA	+	NS	−	2/6	−
Hypo T2 thalamus	−	0/3	−	−	91%	NA	+	NA	NS	3/6	≈57–91%
Hypo T2 of dentate nucleus	−	0/3	−	−	93%	NA	+	NA	NS	−	−
Cerebellar atrophy	−	0/3	−	−	NA	35/43	+	19/19	+	3/6	**≈76%**
Thin corpus callosum	−	0/3	−	−	NA	+ (41/43)	+	19/19	+	2/6	≈61–80%
Bilateral T2 striatal hypersignal	+	+	−	−	−	−	−	−	−	−	3%

*Note:* The Bold values signify the most prevalent signs in *POLR3A*‐related LD.

Abbreviations: F: female; M: male; NA: not available; NS: not specified; +: present; −: absent.

Analysis of the leukoencephalopathy panel data of four patients revealed the same homozygous missense variant NM_007055.4: c.2011T>C; p.(Trp671Arg) in exon 15 of *POLR3A* (10q22.3) that has previously been reported as likely pathogenic according to ACMG/AMP Criteria (PS3, PM2, PM3, PP2, and PP3). Screening of this variant by PCR‐sequencing in the two remaining patients confirmed the diagnosis of *POLR3A*‐related leukodystrophy.

## 4 Discussion

In the present study, we identified a total of six unrelated Tunisian families with confirmed *POLR3A*‐related leukodystrophy. All patients displayed a similar clinical phenotype and carried the same homozygous variant, c.2011T>C; p.(Trp671Arg), indicating a potential founder effect.

*POLR3A* variants are recognized to be associated with a phenotype known as 4H‐leukodystrophy which stands for hypomyelination, hypodontia, and hypogonadotropic hypogonadism. As portrayed in our case series, these features are not mandatory and the clinical picture is often a varying combination of suggestive neurological and extra‐neurological signs (Pelletier et al. 2021; Wolf et al. 2014).

On a molecular level, POLIII‐related leukodystrophies are caused by biallelic variants encoding subunits of the enzyme POLIII, one of the three nuclear polymerases, which is essential to cellular processes including transcription, translation, and RNA maturation. However, the pathophysiology of the disease remains unknown, and a model of transgenic mice with a leukodystrophy causing *POLR3A*‐mutation did not develop motor symptoms such as humans with the same mutation.

The age of onset is variable but most cases have an early‐childhood onset. All of our patients developed symptoms after the age of 15 months which is relatively later than other cases reported (Tewari et al. 2018; Wu et al. 2019). Late‐onset cases with a different phenotype have also been reported (Campopiano et al. 2020). Interestingly, only two of our patients had psychomotor delay, unlike other cases reported in the literature (Bernard et al. 2011; Hiraide et al. 2020; Wolf et al. 2014). This could be explained by the onset of symptoms after walking age in our series, as opposed to the other reported cases, suggesting a possible age‐dependent variability.

Cerebellar signs are a key characteristic of the classical *POLR3A* phenotype, observed in up to 96% of cases. These signs were the inaugural manifestation in all our patients identically to the TACH phenotype of patients described for the first time in Quebec, Canada (Bernard et al. 2010).

In previously reported cases, pyramidal signs were present in up to 80% of patients (Bernard et al. 2011), whereas they were noted in one out of six patients in our series. In fact, some authors have suggested that these signs may be less frequent in younger patients (Wolf et al. 2014). In our series, most patients had nystagmus, which was consistent with literature data (77% of cases). However, myopia was observed in only one patient aged 10 years, which contrasts with the literature where it is reported to be present in up to 87% of cases (Table 2). This difference could be due to the young age of our patients, since short‐sight progressively worsens with age and should be sought for in *POLR3A* patients (Thomas and Thomas 2019).

Four of our patients exhibited dystonia, suggesting that its occurrence might be more common than reported in the literature. However, the actual frequency of extra‐pyramidal signs in *POLR3A*‐related leukodystrophy remains unknown. Some cases have documented atypical presentations, where extra‐pyramidal signs are the main clinical feature, even with absence of white matter involvement (Harting et al. 2020; Hiraide et al. 2020). Severe cases of myoclonus have also been associated with other variants including those in the *POLR1C* gene (Kraoua et al. 2019). Therefore, these extra‐pyramidal signs can be valuable for diagnosis purposes.

On the other hand, extra‐neurological signs are also helpful for the diagnostic approach. These include endocrine abnormalities such as short stature, which is present in 50% of patients, and hypogonadotropic hypogonadism usually manifesting as delayed puberty (Pelletier et al. 2021). Low hormonal rates are a useful biomarker in younger patients.

Dental abnormalities were present in most of our patients, which is consistent with existing literature reporting rates of up to 76%. These abnormalities primarily include hypodontia, delayed tooth eruption, oligodontia, and irregular tooth shape or placement (Vanderver et al. 2013).

Hypomyelination, observed in up to 97% of cases, is a key feature on brain imaging, and is usually diffuse, involving U‐fibers and the corpus callosum.

A relative T2 hyposignal (preservation) of the dentate nuclei and anterolateral nuclei of the thalami and the posterior limb of the internal capsule can also be seen (Steenweg et al. 2010).

Despite cerebellar signs being the predominant feature in our patients, cerebellar atrophy was observed in only half of them. Cerebellar atrophy is typically progressive and has been reported in up to 80% of cases according to the literature, although certain authors have suggested that it may be less frequently observed in patients with *POLR3A* variants (Wolf et al. 2014).

Other unusual imaging patterns have been reported such as selective involvement of corticospinal tracts, isolated cerebellar atrophy without hypomyelination, and striatal involvement (Azmanov et al. 2016; Harting et al. 2020; La Piana et al. 2016).

Motor deterioration, observed in two of our patients, is the expected outcome with most patients becoming wheelchair‐bound during follow‐up (Vanderver et al. 2013). Respiratory failure is the major cause of death in *POLR3A* patients and is due to swallowing difficulties which must be detected early‐on (Bernard et al. 2011).

In terms of molecular diagnosis, the variant identified in our patients is a well‐known recessive pathogenic variant, and has been reported twice in the literature, specifically in Mediterranean patients. Daoud et al. identified this variant in a homozygous state in two siblings from southern Tunisia (Daoud et al. 2013) but did not report their clinical features, while Wolf et al. suggested a higher prevalence of *POLR3A* gene variants and a generally more severe disease in Mediterranean patients (Wolf et al. 2014).

Given the presence of this variant in seven out of seven non‐related families, of different geographic origin in Tunisia, we hypothesize a founder effect of this variant. Other variants with a founder effect have been reported with hypomyelinating leukodystrophies in the Tunisian population, such as the *GJC2* variant c.‐167A>G in Tunisian patients with PMLD disease (Kammoun Jellouli et al. 2013), and the *FAM126* variant c.414 + 1G>T in Tunisian patients with hypomyelination and congenital cataract (Kraoua et al. 2021). The Tunisian population has a relatively high rate of consanguinity (ranging from 20.1% to 39.9%) and endogamy (up to 96% in certain groups/regions), which increases the expression of recessive genetic disorders and the proportion of recessive founder alleles (86%) (Romdhane et al. 2012).

## 5 Conclusions

In the present study, all patients with confirmed *POLR3A*‐related leukodystrophy displayed a similar clinical phenotype and carried the same homozygous variant, indicating a potential founder effect. However, further investigations and a larger sample size are necessary to validate this hypothesis, better understand the genetics of hypomyelinating leukodystrophies in Tunisia, and establish genotype–phenotype correlations.

Considering the observed genetic homogeneity among Tunisian *POLR3A* patients, the recurrent variant identified in this study should be specifically targeted in suspected Tunisian cases. This approach would greatly facilitate diagnosis in index patients, carrier screening, and subsequently enable genetic counseling for their families.

## Author Contributions

All authors contributed equally to this publication.

## Conflicts of Interest

The authors declare no conflicts of interest.

## Data Availability

The data that support the findings of this study are available from the corresponding author upon reasonable request.

## References

[mgg370007-bib-0001] Azmanov, D. N. , S. J. Siira , T. Chamova , et al. 2016. “Transcriptome‐Wide Effects of a POLR3A Gene Mutation in Patients With an Unusual Phenotype of Striatal Involvement.” Human Molecular Genetics 25, no. 19: 4302–4314. 10.1093/hmg/ddw263. 27506977

[mgg370007-bib-0002] Bernard, G. , E. Chouery , M. L. Putorti , et al. 2011. “Mutations of POLR3A Encoding a Catalytic Subunit of RNA Polymerase Pol III Cause a Recessive Hypomyelinating Leukodystrophy.” American Journal of Human Genetics 89, no. 3: 415–423. 10.1016/j.ajhg.2011.07.014. 21855841 PMC3169829

[mgg370007-bib-0003] Bernard, G. , I. Thiffault , M. Tetreault , et al. 2010. “Tremor–Ataxia With Central Hypomyelination (TACH) Leukodystrophy Maps to Chromosome 10q22.3–10q23.31.” Neurogenetics 11, no. 4: 457–464. 10.1007/s10048-010-0251-8. 20640464 PMC4147760

[mgg370007-bib-0004] Campopiano, R. , R. Ferese , S. Zampatti , et al. 2020. “A Novel POLR3A Genotype Leads to Leukodystrophy Type‐7 in Two Siblings With Unusually Late Age of Onset.” BMC Neurology 20, no. 1: 258. 10.1186/s12883-020-01835-9. 32600288 PMC7322863

[mgg370007-bib-0005] Daoud, H. , M. Tétreault , W. Gibson , et al. 2013. “Mutations in POLR3A and POLR3B Are a Major Cause of Hypomyelinating Leukodystrophies With or Without Dental Abnormalities and/or Hypogonadotropic Hypogonadism.” Journal of Medical Genetics 50, no. 3: 194–197. 10.1136/jmedgenet-2012-101357. 23355746

[mgg370007-bib-0006] Dorboz, I. , H. Dumay‐Odelot , K. Boussaid , et al. 2018. “Mutation in POLR3K Causes Hypomyelinating Leukodystrophy and Abnormal Ribosomal RNA Regulation.” Neurology. Genetics 4, no. 6: e289. 10.1212/NXG.0000000000000289. 30584594 PMC6283457

[mgg370007-bib-0007] Harting, I. , M. Al‐Saady , I. Krägeloh‐Mann , et al. 2020. “POLR3A Variants With Striatal Involvement and Extrapyramidal Movement Disorder.” Neurogenetics 21, no. 2: 121–133. 10.1007/s10048-019-00602-4. 31940116 PMC7064625

[mgg370007-bib-0008] Hiraide, T. , K. Kubota , Y. Kono , et al. 2020. “POLR3A Variants in Striatal Involvement Without Diffuse Hypomyelination.” Brain & Development 42, no. 4: 363–368. 10.1016/j.braindev.2019.12.012. 31932101

[mgg370007-bib-0009] Infante, J. , K. M. Serrano‐Cárdenas , M. Corral‐Juan , et al. 2020. “POLR3A‐Related Spastic Ataxia: New Mutations and a Look Into the Phenotype.” Journal of Neurology 267, no. 2: 324–330. 10.1007/s00415-019-09574-9. 31637490

[mgg370007-bib-0010] Kammoun Jellouli, N. , I. Hadj Salem , E. Ellouz , et al. 2013. “Molecular Confirmation of Founder Mutation c.‐167A>G in Tunisian Patients With PMLD Disease.” Gene 513, no. 2: 233–238. 10.1016/j.gene.2012.10.070. 23142375

[mgg370007-bib-0011] Kraoua, I. , Y. Bouyacoub , C. Drissi , et al. 2021. “Hypomyelination and Congenital Cataract: Clinical, Imaging, and Genetic Findings in Three Tunisian Families and Literature Review.” Neuropediatrics 52, no. 4: 302–309. 10.1055/s-0041-1728654. 34192786

[mgg370007-bib-0012] Kraoua, I. , A. Karkar , C. Drissi , et al. 2019. “Novel POLR1C Mutation in RNA Polymerase III‐Related Leukodystrophy With Severe Myoclonus and Dystonia.” Molecular Genetics & Genomic Medicine 7, no. 9: e914. 10.1002/mgg3.914. 31368241 PMC6732337

[mgg370007-bib-0013] La Piana, R. , F. K. Cayami , L. T. Tran , et al. 2016. “Diffuse Hypomyelination is Not Obligate for POLR3‐Related Disorders.” Neurology 86, no. 17: 1622–1626. 10.1212/WNL.0000000000002612. 27029625 PMC4844237

[mgg370007-bib-0014] Macintosh, J. , S. Perrier , M. Pinard , et al. 2023. “Biallelic Pathogenic Variants in POLR3D Alter tRNA Transcription and Cause a Hypomyelinating Leukodystrophy: A Case Report.” Frontiers in Neurology 14: 1254140. 10.3389/fneur.2023.1254140. 37915380 PMC10616956

[mgg370007-bib-0015] Nikkhah, A. , and S. Rezakhani . 2022. “Developmental Regression and Movement Disorder as a Phenotypic Variant of POLR3A Mutation‐Case Report.” Clinical Case Reports 10, no. 11: e6556. 10.1002/ccr3.6556. 36397839 PMC9664542

[mgg370007-bib-0016] Pelletier, F. , S. Perrier , F. K. Cayami , et al. 2021. “Endocrine and Growth Abnormalities in 4H Leukodystrophy Caused by Variants in POLR3A, POLR3B, and POLR1C.” Journal of Clinical Endocrinology and Metabolism 106, no. 2: e660–e674. 10.1210/clinem/dgaa700. 33005949 PMC7823228

[mgg370007-bib-0017] Romdhane, L. , R. Kefi , H. Azaiez , N. Ben Halim , K. Dellagi , and S. Abdelhak . 2012. “Founder Mutations in Tunisia: Implications for Diagnosis in North Africa and Middle East.” Orphanet Journal of Rare Diseases 7: 52. 10.1186/1750-1172-7-52. 22908982 PMC3495028

[mgg370007-bib-0018] Saitsu, H. , H. Osaka , M. Sasaki , et al. 2011. “Mutations in POLR3A and POLR3B Encoding RNA Polymerase III Subunits Cause an Autosomal‐Recessive Hypomyelinating Leukoencephalopathy.” American Journal of Human Genetics 89, no. 5: 644–651. 10.1016/j.ajhg.2011.10.003. 22036171 PMC3213392

[mgg370007-bib-0019] Shimojima, K. , S. Shimada , A. Tamasaki , et al. 2014. “Novel Compound Heterozygous Mutations of POLR3A Revealed by Whole‐Exome Sequencing in a Patient With Hypomyelination.” Brain & Development 36, no. 4: 315–321. 10.1016/j.braindev.2013.04.011. 23694757

[mgg370007-bib-0020] Steenweg, M. E. , A. Vanderver , S. Blaser , et al. 2010. “Magnetic Resonance Imaging Pattern Recognition in Hypomyelinating Disorders.” Brain 133, no. 10: 2971–2982. 10.1093/brain/awq257. 20881161 PMC3589901

[mgg370007-bib-0021] Tewari, V. V. , R. Mehta , C. M. Sreedhar , et al. 2018. “A Novel Homozygous Mutation in POLR3A Gene Causing 4H Syndrome: A Case Report.” BMC Pediatrics 18, no. 1: 126. 10.1186/s12887-018-1108-9. 29618326 PMC5883641

[mgg370007-bib-0022] Thomas, A. , and A. K. Thomas . 2019. “POLR3‐Related Leukodystrophy.” Journal of Clinical Imaging Science 9: 45. 10.25259/JCIS_116_2019. 31768296 PMC6826334

[mgg370007-bib-0023] Vanderver, A. , D. Tonduti , G. Bernard , et al. 2013. “More Than Hypomyelination in Pol‐III Disorder.” Journal of Neuropathology and Experimental Neurology 72, no. 1: 67–75. 10.1097/NEN.0b013e31827c99d2. 23242285 PMC3797528

[mgg370007-bib-0024] Wolf, N. I. , A. Vanderver , R. M. L. van Spaendonk , et al. 2014. “Clinical Spectrum of 4H Leukodystrophy Caused by POLR3A and POLR3B Mutations.” Neurology 83, no. 21: 1898–1905. 10.1212/WNL.0000000000001002. 25339210 PMC4248461

[mgg370007-bib-0025] Wu, S. , Z. Bai , X. Dong , et al. 2019. “Novel Mutations of the POLR3A Gene Caused POLR3‐Related Leukodystrophy in a Chinese Family: A Case Report.” BMC Pediatrics 19, no. 1: 289. 10.1186/s12887-019-1656-7. 31438894 PMC6704677

